# GH10 xylanase D from *Penicillium funiculosum*: biochemical studies and xylooligosaccharide production

**DOI:** 10.1186/1475-2859-10-20

**Published:** 2011-04-05

**Authors:** Mickael Lafond, Alexandra Tauzin, Véronique Desseaux, Estelle Bonnin, El-Hassan Ajandouz, Thierry Giardina

**Affiliations:** 1 Université Paul Cézanne, ISM2/BiosCiences UMR CNRS 6263 (Université Aix Marseille III/CNRS), service 342, Faculté des Sciences et Techniques Saint-Jérôme, 13397 Marseille Cedex 20, France; 2 INRA, UR 1268 - Biopolymères - Interactions - Assemblages, 44316 Nantes Cedex 03, France

**Keywords:** xylanase, GH10, Penicillium funiculosum, xylooligosaccharides

## Abstract

**Background:**

The filamentous fungus *Penicillium funiculosum *produces a range of glycoside hydrolases (GH). The *XynD *gene, encoding the sole *P. funiculosum *GH10 xylanase described so far, was cloned into the pPICZαA vector and expressed in methylotrophe yeast *Pichia pastoris*, in order to compare the results obtained with the *P. funiculosum *GH11 xylanases data.

**Results:**

High level expression of recombinant XynD was obtained with a secretion of around 60 mg.L^-1^. The protein was purified to homogeneity using one purification step. The apparent size on SDS-PAGE was around 64 kDa and was 46 kDa by mass spectrometry thus higher than the expected molecular mass of 41 kDa. The recombinant protein was N- and O-glycosylated, as demonstrated using glycoprotein staining and deglycosylation reactions, which explained the discrepancy in molecular mass. Enzyme-catalysed hydrolysis of low viscosity arabinoxylan (LVAX) was maximal at pH 5.0 with *K*m_(app) _and *k_cat_*/*K*m_(app) _of 3.7 ± 0.2 (mg.mL^-1^) and 132 (s^-1^mg^-1^.mL), respectively. The activity of XynD was optimal at 80°C and the recombinant enzyme has shown an interesting high thermal stability at 70°C for at least 180 min without loss of activity. The enzyme had an endo-mode of action on xylan forming mainly xylobiose and short-chain xylooligosaccharides (XOS). The initial rate data from the hydrolysis of short XOS indicated that the catalytic efficiency increased slightly with increasing their chain length with a small difference of the XynD catalytic efficiency against the different XOS.

**Conclusion:**

Because of its attractive properties XynD might be considered for biotechnological applications. Moreover, XOS hydrolysis suggested that XynD possess four catalytic subsites with a high energy of interaction with the substrate and a fifth subsite with a small energy of interaction, according to the GH10 xylanase literature data.

## 1. Introduction

Xylanases (endo-1,4-β-xylanases; EC 3.2.1.8) catalyze the hydrolysis of β-1,4 bonds of xylan, the major hemicellulose component of the plant cell wall. According to the sequence-based glycoside hydrolase (GH) classification (CAZy classification) [1], xylanases are mainly grouped into families 10 (GH10) and 11 (GH11) but they were also found in glycoside hydrolase families 5, 8 and 43 http://www.cazy.org. A GH7 enzyme from *Penicillium funiculosum *with xylanase/cellobiohydrolase activities was also reported and called XynA. GH10 family contains plant, fungal and bacterial enzymes whereas the structurally unrelated GH11 family only includes fungal and bacterial enzymes [1-3]. Xylanases are used in many food and feed sectors such as additives for broiler feed, bread making, in separation processes of gluten from starch in wheat or other cereals gluten from starch, production of juice from fruits or vegetables, extracting more fermentable sugar from barley for brewing, etc [4-6]. Recently, the potential applications of xylanases for bioconversion of hemicellulosic materials to xylo-oligosaccharides (XOS) have gained popularity instead of acid hydrolysis which is not environmentally feasible [7-9]. XOS have been shown to impact on human health by reducing cholesterol level and precancerous lesions, improving the biological availability of calcium and exhibiting a positive effect on gut microbiota [10-12]. The latter positive effect should be due to the enhancement of the production of different prebiotic molecules like short chain fatty acids known to promote the growth of beneficial bacteria such as *Lactobacillus or Bifidobacteria *and to lowering the cecal pH value [13-15].

In addition to the health promoting effects, XOS are moderately sweet and stable over a wide range of pH and temperatures and have organoleptic characteristics that make them suitable for incorporation into foods [16,17].

Lignocellulosic biomass degradation used for biotechnological XOS production, needs thermal treatments coupled to thermostable xylanases. Moreover, a large amount of hemicellulosic fraction is insoluble and need action of appropriate enzymes. So, researches concerning new xylanases acting on high temperatures and able to hydrolyze insoluble substrate remain topical.

The filamentous fungi *Penicillium funiculosum *secretes a lot of glycosidases and the recent sequencing of the *P. funiculosum *genome, allowed discriminating about 200 genes encoding proteins with potential glycosidase activity [18]. Among the glycosidases which could be used during biotechnological processes, different types of xylanases were purified and their genes have been cloned [19,20]. XynB (GH11) and XynC (GH11) were recently extensively studied [21,22] whereas little is known concerning XynD the only GH10 member family found to date in this microorganism [20]. GH10 xylanases typically exhibit a molecular weight ≥30 kDa and a low p*I *[23]. The crystal structures of several GH10 enzymes showed that the catalytic domain is an 8-fold α/β-barrel forming a 'salad bowl'. Often, one or more extra domains are present, corresponding to carbohydrate binding module (CBM1) which binds to cellulose [24].

The activity of GH10 xylanases on AXs produces shorter XOS than those produced by GH11 xylanases. The former can act near the substituted xylose residue whereas the latter are bothered by additional groups like 4-O-methyl-D glucuronate, acetate and α-L-arabinofuranose, thus restricting the access to the β-1,4-linkages in the xylan backbone [25]. It is worth noting that in the GH10 family, the members are able to produce different end-products due to the difference in their catalytic site [26].

Here we report the molecular characterization of XynD from *Penicillium funiculosum *encoding a 41 kDa GH10 xylanase (XynD). The cDNA cloning and heterologous expression of XynD in *Pichia pastoris *was performed, and biochemical properties of the recombinant protein were determined, notably showing a natural thermal resistance. The enzyme was also characterized under kinetic viewpoint using various short and long substrates, especially regarding the XOS liberation profile.

## 2. Materials and methods

### Materials

The pPICZαA expression vector and the *P. pastoris *expression kit used, including the *P. pastoris *strain X-33, zeocin, oligonucleotides and all restriction DNA modifying enzymes (except DNA polymerase) were from Invitrogen (Groningen, Netherlands). PrimeSTAR™ HS DNA polymerase for polymerase chain reactions (PCR) was from Takara (Madison, USA). Escherichia coli DH5α (*sup*E44, *hsd*R17, *rec*A1, *end*A1, *gyr*A96, *thi*-1, and *rel*A1) was used for the DNA procedures (Invitrogen). Sodium phosphate and citric acid was from Sigma-Aldrich and Sephacryl S200 HR 26/60 chromatographic columns were from Amersham-Pharmacia Biotech (Uppsala, Sweden). Ultracel™ system and Ultracel™ PES membrane were from Millipore (Billerica, USA). The XynD concentration was determined by the Bradford method (1976) using the "Protein Assay" Reagent from Bio-Rad (Marnes-La-Coquette, France) [27].

### Cloning, expression and purification of XynD

cDNA encoding XynD was synthesized by Geneart (Germany) using the GenBank AJ634957.1 accession number. The pPICZαA-derived *Pichia pastoris *expression plasmid with the cDNA insert encoding XynD was constructed using standard procedures. The insert was purified using the QIAquick^® ^(QIAGEN) purification kit and ligated into the XhoI/XbaI pPICZαA plasmid, which was digested with PmeI to linearize the DNA for integration into *P. pastoris *genome. The linearized DNA was used for electroporation into *P. pastoris *strain X-33 using a Multiporator^® ^(Eppendorf) at 1500 V for 5 ms. The transformants were selected using Minimal Methanol and Minimal Dextrose plates. Finally, in order to select multicopy vector strain transformants YPDS plates containing 100, 200, 500 and 1000 μg/mL zeocin^® ^were used.

Large-scale expression was carried out as previously described [21]. The culture supernatant was concentrated using Ultracel™ PES ultrafiltration membrane (3 kDa) and subjected to gel filtration at a flow rate of 1.5 mL.min^-1 ^on a Sephacryl S-200 column connected to FPLC^® ^equipment (Akta Purifier 10, GE Healthcare, Piscataway, USA) and eluted with 50 mM Na-phosphate buffer, pH 7.2, containing 150 mM NaCl. The fractions containing xylanases activity were pooled.

### Polyacrylamide gel electrophoresis, glycosylation staining, N-terminal sequencing, molecular mass determination, and mass spectrometry analysis

SDS-PAGE was performed in 10 or 12% (w/v) polyacrylamide gel in the presence of 2% SDS under reducing or non-reducing conditions [28]. Proteins were visualized by Coomassie blue R-250 staining. For glycosylation determination, SDS- polyacrylamide gel were stained for carbohydrate with periodic acid-Schiff (PAS) using a SIGMA glycoprotein staining kit^® ^according to the manufacturer's protocol.

N-terminal amino acid sequencing of the Ponceau red stained protein after electro-transfer on a polyvinylidene difluoride membrane was performed by automated repetitive Edman degradation on a model 494 Procise™ Protein Sequencer (Applied Biosystems Division) [29]. Molecular mass determination was performed by Matrix-assisted laser desorption ionization/mass spectrometry (Ettan Maldi-Tof Pro, GE Healthcare, Uppsala, Sweden) as previously described [21].

### Deglycosylation assay

Purified XynD was N-deglycosylated using endo-β-acetylglucosaminidase H (Endo-H, Sigma Chemical Co.) and/or α-mannosidase from *Canavalia ensiformis *(Sigma Chemical Co.). Treatment with Endo-H was carried out in a 10 mM sodium citrate buffer, pH 5.0, for 6 h at 37°C, with a ratio of 1000 U: 77.5 μg native and denaturated enzyme following the supplier recommendations. For O-deglycosylation, N-deglycosylated XynD was treated with α-mannosidase in a 50 mM sodium phosphate buffer, pH 6.0 for 16 h at 37°C, with a ratio of 14.2 U: 60 μg protein.

### Enzyme assays and effect of temperature and pH on enzyme activity

Xylanase activity was measured using the dinitrosalicylic acid (DNS) assay. Aliquot of the xylanase (10 μL) was mixed with 10 mg.mL^-1 ^low viscosity AX from wheat (LVAX) (Megazyme) in McIlvaine's buffer, pH 5.0 (total volume reaction volume 250 μl) at 70°C for 2 min. The reaction was stopped by the addition of 250 μl DNS reagent before boiling the mixture during 5 min. The reactions were cooled and centrifuged for 3 min at 17 000 *g *and 200 μL was transferred to a microplate and the absorption was measured at 545 nm. One unit (U) of xylanase activity was defined as the amount of protein that released 1 μmol of xylose min^-1^, based on xylose calibration curve. Optimal pH for xylanase activity was estimated using the DNS assay with LVAX (10 mg.mL^-1^) in McIlvaine's buffer in a pH range of 2.0 to 8.0. Optimal temperature was also estimated using the same xylanase assay with LVAX (10 mg.mL^-1^) in McIlvaine's buffer at pH 5.0 and temperatures ranging from 20°C to 100°C. Thermal stability was performed at pH 5.0 and 70°C or 80°C for different time periods before performing activity assay.

### Determination of substrate specificity and kinetic parameters

The substrate specificity of purified recombinant XynD was evaluated using the following substrates at 10 mg.mL^-1 ^in McIlvaine's buffer, pH 5.0: LVAX, medium viscosity AX (MVAX, Megazyme), high viscosity AX (HVAX, Megazyme), water-insoluble AX (IAX, Megazyme), Birchwood Xylan (Sigma), Beechwood Xylan (Sigma), xyloglucan from tamarind (XGT, Megazyme). Water-extractable wheat AX with different ratio arabinose:xylose were prepared by sequential precipitation with 30%, 50% and 60% ethanol (AXMF30, AXMF50, AXMF60), [30]. Their A/X values were 0.33, 0.53 and 0.73, respectively. Activity was measured by the DNS assay as described above.

Determinations of kinetic parameters were realized using the same condition described previously with LVAX as the substrate. Six concentrations of LVAX (2.5-20 mg.mL^-1^) were used. The final concentration of XynD was 1.6 nM. The reaction was stopped at appropriate time intervals (2, 4, 6, 8 and 10 min) and the initial rates of hydrolysis of LVAX have been determined accordingly and expressed as μM xylose/min. All experiments were repeated three times. Kinetic analyses were performed using the Michaelis-Menten, Lineweaver-Burk, Eadie-Hofstee and Dixon plots.

### XOS analysis and quantification

Xylooligosaccharides and their products generated after XynD hydrolysis were analysed by high performance anion exchange chromatography coupled with a pulsed amperometric detection (HPAEC-PAD; Dionex, Sunnyvale, CA, USA) equipped with a Carbo-Pac PA-100 analytical column (250×4 mm), a GP40 gradient pump, and a AS3500 auto-sampler (Thermo-Electron). The protocol used is the same as described by Cervera-Tison *et al*, in 2009 excepted for the composition of buffer A (NaOAC, 5 mM; NaOH, 80 mM) [31]. To evaluate the activity of the XynD on XOS (xylohexaose, xylopentaose, xylotetraose and xylotriose), initial slopes of progress curves were used to determine the catalytic efficiency (*k*_cat_/*K*m) of the reaction following the equation of Matsui as previously described by Cervera-Tison *et al*, in 2009 [31]. All assays were carried out in duplicate.

## 3. Results and discussion

### Characterization of *XynD *gene

**Figure 1:**
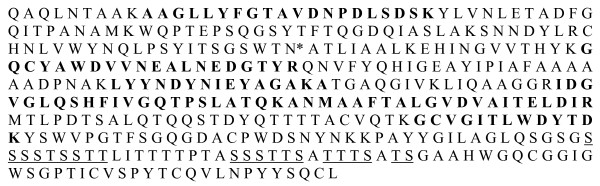
**XynD peptidic sequence**. N*: N-glycosylation putative site, ST: O-glycosylation putative sites are underline. Identified peptides of N- and O-deglycosylated protein after tryptic digestion and mass spectrometry appear in bold.

The *XynD *cDNA is predicted to encode a modular mature protein of 390 amino acid residues, which includes a 317 amino acid catalytic module, a 38 amino acid Ser/Thr rich linker and a 35 amino acid carbohydrate binding module with significant similarity to the carbohydrate binding module characteristic of fungal CBM1 [24]. Deduced molecular mass and pI of XynD were calculated to be 41540 Da and 5.02, respectively. One N-glycosylation site was detected, at position N101, using NetNGlyc 1.0 Server and the DictyOGlyc server proposed several potential O-glycosylation sites (Figure 1).

A BLAST search showed that XynD shares highest amino acid sequence identity of 87 and 86% with family 10 xylanases from *Talaromyces stipitatus *and *Penicillium marneffei*, respectively (data not shown). This result together with biochemical characteristics, suggested that XynD, belongs to GH10 family, confirming the results from Furniss *et al*. in 2005 [20].

### Expression, purification and biochemical characterization of XynD

In order to study the biochemical properties of the xylanase from *P. funiculosum*, the *XynD *cDNA was cloned into the pPICZαA plasmid and expressed in *Pichia pastoris *as a recombinant protein using the native *Saccharomyces cerevisiae *α-factor secretion signal sequence under the control of the AOX1 promoter. After an induction step with methanol, a major protein band with an apparent molecular mass of 64 kDa, which was not present in the supernatant of untransformed cells, was observed in the culture filtrate after SDS-PAGE analysis (data not shown). The amount of the 64 kDa protein increased with time of induction (data not shown). Only trace amounts of other proteins were present in the culture supernatant. XynD was purified to homogeneity as described in the Materials and Methods section, giving a single peak after chromatography on a Sephacryl S200 gel containing 21% of the supernatant activity. Thus, the enzyme was purified 1.9 fold with a specific activity of 33.9 U.mg^-1^.

**Figure 2:**
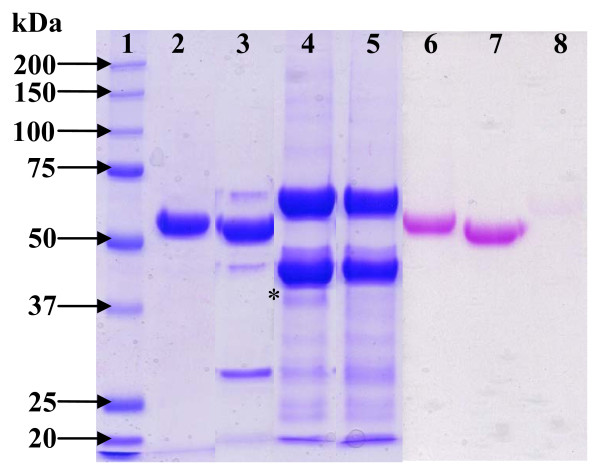
**SDS-PAGE (10%) of XynD and deglycosylated XynD (1-5: Coomassie R250 coloration, 6-8: Schiff reagent coloration)**. (1) Molecular weight; (2) Native XynD; (3) XynD N-deglycosylated; (4) *: XynD N- and O- deglycosylated; (5) α-mannosidase; (6) Native XynD; (7) XynD N-deglycosylated; (8) XynD N- and O-deglycosylated.

The N-terminal sequence EAEAQAQ was in complete agreement with that expected from the recombinant protein, EAEA corresponding to the C-terminal end of the α-factor signal peptide of pPICZαA vector after efficient peptidase signal digestion (Kex2), followed by QAQ from XynD N-terminal mature sequence. The purified recombinant enzyme migrated in SDS-PAGE give a single band around 64 kDa (Figure 2, lane 2). The estimated size on electrophoresis gel and the 46 kDa molecular mass determinate by mass spectrometry, thus much higher than the theoretical size of 41540 Da, suggesting that the recombinant XynD was glycosylated. The presence of sugars linked to the protein was confirmed by periodic acid Schiff staining (Figure 2, lane 6). One N-glycosylation site is present in XynD amino acid sequence at position N101, suggesting that N-glycosylation is responsible for the higher molecular mass. To confirm this change was due to glycosylation, deglycosylation analysis was performed. After treatment with Endo H to remove N-linked carbohydrate moieties, only a single band located at about 60 kDa was observed (Figure 2, lane 3) which confirms that XynD was N-glycosylated. However, the molecular mass was still higher than that expected and periodic acid Schiff staining still positive, suggesting the presence of O-glycosylated moieties (Figure 2, lane 7). Actually, linker peptide connecting catalytic and carbohydrate-binding modules in fungal glycoside hydrolases are rich in Ser and Thr residues, and they are often *O*-glycosylated [32].

Thus, α-mannosidase treatment was performed to remove O-glycosylated sugars and a band with a molecular mass around 43 kDa appeared, corresponding to theoretical molecular mass of XynD and the deglycosylated enzyme was not stained by periodic acid Schiff (Figure 2, lane 4 and 8). Tryptic digestion and mass spectrometry analysis of the N- and O-deglycosylated 43 kDa protein confirmed that enzyme is XynD (Figure 1). A large amount of polymeric α-mannosidase which gave 2 different bands around 44 and 66 kDa (Figure 2, Lane 5) was necessary to totally O-deglycosylated XynD.

The experimental pI determined by isoelectrofocusing was 5.9 (data not shown). This result and the experimentally determined molecular mass were in good agreement with data collected in the literature. It is worth noting that almost all basic xylanases having a molecular mass of less than 30 kDa belong to GH11 family, whereas acid xylanases have a molecular mass higher than 30 kDa and belong to GH10 family [33].

### Effect of temperature and pH on XynD activity

**Figure 3:**
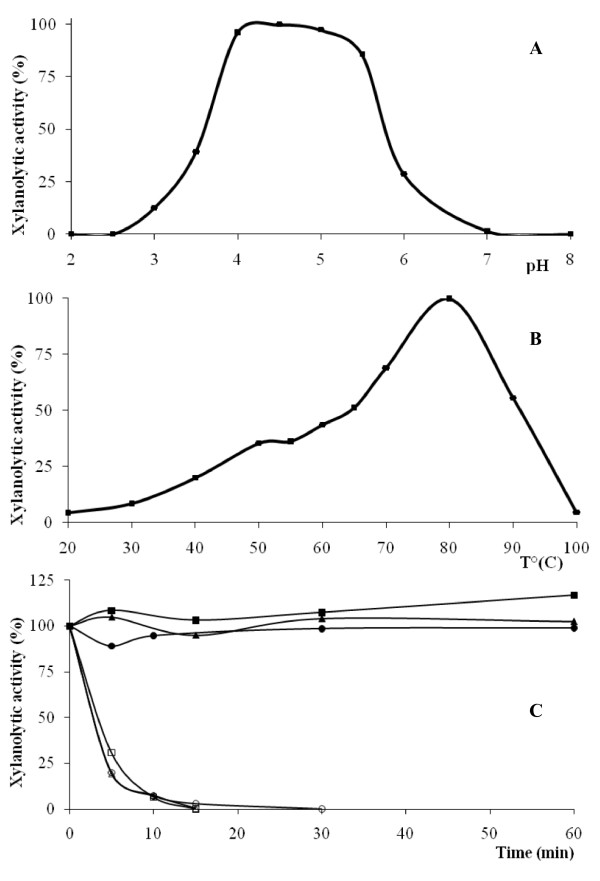
**pH (A) and temperature (B) optima and thermostability (C) of XynD from *P. funiculosum***. (A) Effect of pH on the activity. Enzyme activity was assayed in a pH range of 2.0-8.0. (B) Effect of temperature on the activity. Enzyme activity was assayed at various temperatures of 20-100°C in 100 mM McIlvaine's buffer (pH 5.0). (C) Thermal stability of the enzyme. Thermal stability of the enzyme native (square) N-deglycosylated (triangle), N- and O-deglycosylated (circle) at 70°C (full symbol) and 80°C (empty symbol) were measured after incubation during several minutes at pH 5.0.

The effects of pH and temperature on the enzymatic activity were investigated on the recombinant enzyme. XynD displayed optimum activity in the 4.0 to 5.5 pH range whereas the activity was lost at pH 2.5 (Figure 3A). At pH 5.0, the optimal enzymatic activity was observed at 80°C whereas the activity drastically decreased above 90°C (Figure 3B). The thermal stability of the enzyme was determined at 70 and 80°C at the same pH value (Figure 3C). XynD enzymatic activity decreases about 80% after 10 min at 80°C in contrast XynD is stable at least one hour at 70°C. The high stability of XynD at 70°C should be highly useful for industrial purposes. This could be due to post-translational modifications of xylanase during excretion process, such as glycosylation. However, deglycosylated enzyme treated as above was found to be as stable as the glycosylated enzyme, suggesting that glycosylation had no effect on thermal stability.

### Substrate specificity of XynD from *P. funiculosum*

The hydrolytic activity of XynD from *P. funiculosum *on various substrates was determined (Table 1). The enzyme showed a high specificity and a insensitivity towards different soluble xylans tested with specific activities ranging between 50.5 ± 0.5 U.mg^-1 ^for Birchwood Xylan and 114.3 ± 1.0 U.mg^-1 ^for HVAX. The rate of insoluble xylan degradation was similar to that of soluble xylan degradation and XynD seemed to be a few sensitive to the xylan substitution degree with specific activities ranging between 80.6 ± 1.8 U.mg^-1 ^for AXMF60 and 120.8 ± 1.6 U.mg^-1 ^for AXMF30. The equivalent results were previously observed for the GH10 xylanase from *Aspergillus aculeatus *[34]. Because it is well-known that GH10 xylanases are preferentially active against soluble substrates, this result is very interesting, suggesting an important role of the CBM to hydrolyze the insoluble and viscous xylans during XynD action [35]. This would be highly useful for degradation of cell wall polysaccharides allowing easier access to starchy materials in cereals or other starch-rich grains. Finally, low activity was detected using xyloglucan as substrate.

**Table 1:** Specific activities (U.mg^-1^) of XynD on different substrates.

Substrates	Specific Activity (U.mg^-1^)	SD
*LVAX*	97.9	8.5
*MVAX*	106.4	0.2
*HVAX*	114.3	1.0
*IAX*	85.7	5.7
*Birchwood X*	50.5	0.5
*Beechwood X*	60.1	0.5
*AXMF30*	120.8	1.6
*AXMF50*	89.4	2.8
*AXMF60*	80.6	1.8
*XGT*	0.9	0.1

SD: standard deviation

### Determination of kinetic parameters

The XynD-catalysed hydrolysis of LVAX was measured with the optimal temperature (70°C) and pH 5 conditions (Table 2). The experimental initial rates were used for determination of *k*_cat _and *K*m_(app) _and calculation of the catalytic efficiency, *k*_cat_/*K*m_(app) _according the kinetic models of Michaelis-Menten, Lineweaver-Burk and Eadie-Hofstee (plot not shown). The *K*m_(app) _of XynD with LVAX as the substrate is 3 to 8 fold higher than those of *P. funiculosum *GH11 xylanases but the catalytic efficiency (*k*_cat/_*K*m_(app)_) were similar [21,22].

**Table 2:** Kinetic values for XynD with LVAX as substrate and comparison with the *P. funiculosum *family 11 xylanases.

Enzyme	Plot	*K*_m(app)_	*k*_cat_	*k_cat_/K*_m(app)_	References
		(mg.mL^-1^)	(s^-1^)	(s^-1^mg^-1^.mL)	
*XynD*	M.-M.	3.7 ± 0.2	399	108	This study
*XynD*	L.-B.	5.0 ± 0.2	833	167	"
*XynD*	E.-H.	3.9 ± 0.2	523	134	"
*XynB*	M.-M.	40.0 ± 3.0	5405	135	[21]
*XynC*	M.-M.	14.4 ± 0.7	2939	204	[22]

For XynD, the parameters were determined from primary plots of Michaelis-Menten (M.-M), Lineweaver-Burk (L.B) and Eadie-Hofstee (E.-H) plots.

**Figure 4:**
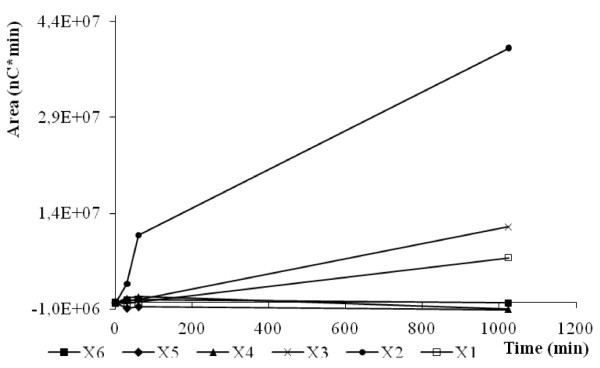
**HPAEC analysis of wheat LVAX hydrolysis by XynD**. XynD (25.0 nM) was incubated with 5 mg.mL^-1 ^wheat AX in McIlvaine's buffer of pH 5.0 at 50°C. The reaction was stopped after 0, 30, 60 and 1024 min and subjected to HPAEC analysis. Xylose (X1), xylobiose (X2), xylotriose (X3), xylotetraose (X4), xylopentaose (X5), and xylohexaose (X6).

In order to evaluate the mode of action of XynD, the products generated using highly polymeric substrates and on short substrates ranging from xylotetraose to xylohexaose were analysed by HPAEC-PAD. XynD displayed mainly an endo-activity against wheat AX (LVAX) because the XOS generated during the initial stages of hydrolysis were progressively degraded yielding principally xylobiose and to a lower extent of xylotriose and xylose (Figure 4). This characteristic is obviously similar to GH11 xylanases that yield xylobiose and xylotriose as main end reaction products [22,31].

**Figure 5:**
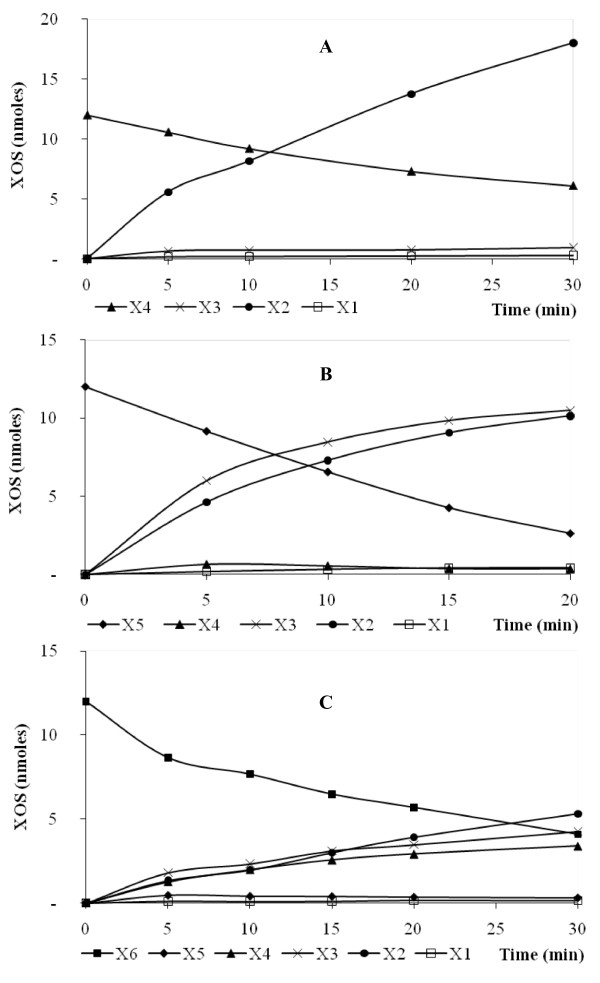
**Progress curves of the XOS generated by XynD after hydrolysis of xylotetraose (A), xylopentaose (B), and xylohexaose (C)**. Recombinant enzymes were incubated with 100 μM of XOS in McIlvaine's buffer pH 5.0 at 70°C. The quantity of xylose (X1), xylobiose (X2), xylotriose (X3), xylotetraose (X4), xylopentaose (X5), and xylohexaose (X6) produced during the course of the reaction is indicated. The concentrations of enzymes used were 640 nM (A), 16.1 nM (B), and 6.4 nM (C).

XynD exhibited an exclusive endo-mode of action against XOS. Indeed, xylohexaose was hydrolysed to xylotriose, xylotetraose and xylobiose; xylopentaose to xylobiose and xylotriose; whereas xylotetraose was cleaved to produce exclusively xylobiose (Figure 5). No significant amount of xylose was generated during hydrolysis of XOS. The catalytic efficiency (*k*_cat_/*K*m) increased slightly with increasing chain length of XOS up to n = 6 (Table 3) and the relative value on xylotetraose, xylopentaose and xylohexaose, was 1:1.3:8.8. Xylanase C from *P. funiculosum*, a GH11 xylanase family, showed different catalytic efficiency (*k*_cat_/*K*m) results on the same substrates with the following relative values: 1:2.1:39.3 [22]. Finally, the most important difference was found with XynB from *P. griseofulvum *1:12:129 [31]. Both the studies concluded that these xylanases potentially contain six subsites in their active site. Xylohexaose degradation produced only xylotriose, indicating that the minimum length of the substrate is four residues. Moreover, the small difference of the XynD catalytic efficiency against xylotetraose and xylopentaose and against xylopentaose and xylohexaose suggest that XynD possess four catalytic subsites with a high energy of interaction with the substrate and a fifth subsite with a small energy of interaction. However this hypothesis will be confirmed by a modeling study.

**Table 3:** Catalytic efficiencies of XynD compared to 3 *Penicillium sp. *GH11 xylanases.

Enzymes	Substrates	*k*_cat_/*K*m	References
	(100 μM)	(min^-1^.M^-1^)	
*XynD*	X6	5.9.10^6	This study
	X5	4.4.10^6	"
	X4	1.5.10^6	"
*XynC*	X6	6.5.10^6	[22]
	X5	3.1.10^6	"
	X4	1.8.10^5	"
*XynA*	X6	2.6.10^6	[22]
	X5	4.6.10^5	"
	X4	3.0.10^4	"
*XynB*	X6	8.0.10^5	[31]
	X5	7.4.10^4	"
	X4	6.2.10^3	"

Xylohexaose (X6), xylopentaose (X5) and xylotetraose (X4) were used as substrates.

## 4. Conclusion

Filamentous fungus *P. funiculosum *is known for its capacity to produce xylanases with different capacities that may provide the fungus with the maximum potential to degrade xylans from different sources. In this study, the *P. funiculosum *GH10 unique gene encoding a xylanase was cloned and express in *P. pastoris *yeast. Biochemical characterization and enzymatic analysis showed that XynD presented a high temperature optimal for hydrolysis activity, was thermostable and it was a versatile xylanase in term of activities and catalytic efficiencies than the others *P. funiculosum *xylanases. Because of its attractive properties XynD might be considered for biotechnological applications.

## Abbreviations

AX: arabinoxylan; AXMF: AXs with different ratio A/X; CBM: carbohydrate binding module; DNS: dinitrosalicylic acid; GH: glycoside hydrolase; HAVX: high viscosity AXs; HPAEC-PAD: high performance anion exchange chromatography; IAX: insoluble AXs; LVAX: low viscosity AXs; MAVX: medium viscosity AXs; PAS: periodic acid Schiff; PBS: phosphate-buffered saline; PES: polyether sulfone; XGT: xyloglucan; XOS: xylooligosaccharides.

## Competing interests

The authors declare that they have no competing interests.

## Authors' contributions

ML was involved in study design, data interpretation and manuscript writing; AT was involved in biochemical and enzymatic analysis; VD was involved in biochemical analysis design and data interpretation; EB was involved in the determination of the activity assays and in the data interpretation; EHA was involved in data interpretation and manuscript writing; TG was the coordinator of the study design and was involved in data interpretation and manuscript writing and editing. All authors read and approved the final manuscript.
